# Editorial: Primary Glial and Immune Cell Pathology in Neurodegenerative Diseases

**DOI:** 10.3389/fneur.2021.765376

**Published:** 2021-10-18

**Authors:** András Lakatos, Gabor Petzold

**Affiliations:** ^1^John van Geest Centre for Brain Repair, Department of Clinical Neurosciences, University of Cambridge, Cambridge, United Kingdom; ^2^Wellcome Trust-MRC Cambridge Stem Cell Institute, Cambridge, United Kingdom; ^3^German Center for Neurodegenerative Diseases (DZNE), Bonn, Germany; ^4^Division of Vascular Neurology, University Hospital Bonn, Bonn, Germany

**Keywords:** glia, astrocyte, microglia, immune cell, neurodegeneration, Alzheimer's disease, Parkinson's disease, glaucoma

Non-neuronal cells in the brain have been proposed as key modulators of neuronal network function in health and in neurological disease. Until recently, the involvement of glial cells—including astrocytes, microglial cells, oligodendrocytes and their progenitors—and infiltrating immune cells in neurodegenerative diseases has been merely viewed as a secondary adaptive response to disease-specific neuronal pathology. It emerges that glial and other non-neuronal cells can also be directly affected by neurodegenerative cues, worsening neuronal dysfunction (1, 2). This has highlighted new potential avenues in targeting disease pathways in a broad spectrum of neurodegenerative conditions, which would require teasing apart primary pathology from secondary processes in multiple cell types.

In this issue we brought together some of the leaders in glial cell biology and neurodegenerative disease research to cover recent advances in pathomechanistic studies informing novel experimental treatment strategies in six review articles.

The Research Topic starts with two articles discussing the emerging role of astrocytes in neurodegeneration. One of the most argued, and therapeutically relevant issues, is the extent to which morphological, gene expression and signaling changes characterize a primary cell-autonomous astrocyte pathology or a secondary adaptive or “reactive” response to damage. There is a consensus in the field to steer away from the less reliable phenotypic classifications and rather assess the function of various astrocytic states in individual disease models to help reveal potential treatment targets (3). What complicates this assessment is the growing evidence that astrocytes already represent a large and heterogenous cell population (4, 5). Monterey et al. review the “many faces” of astrocytes, which can represent both a regional cell diversity and a broad functional spectrum in disease with multiple targetable elements. They discuss recent advances in sequencing technologies, and how they can be used to distinguish pathological cell states and signaling disturbances in Alzheimer's disease (AD). The article by García-Bermúdez et al. emphasizes that glaucoma, a common eye disease that damages the optic nerve and the retina, shares glia-mediated mechanisms with many neurodegenerative diseases, such as AD. The concept of common pathological processes between the eye and the brain is also supported by observations of retinal changes in AD patients, forming the basis of an emerging ophthalmological diagnostic opportunity for neurodegenerative disorders (6). Furthermore, the authors provide a broad overview on retinal glia-ganglion cell interactions as potential therapeutic targets. Altogether, these articles highlight the importance of distinguishing homoeostatic and detrimental astrocyte responses for identifying potentially targetable pathological signaling cascades.

Another major advance concerns microglial transcriptomic and inflammatory signaling changes in neurodegenerative pathologies. Similar to astrocytes, microglia may adapt to neuronal injuries by regaining their homeostatic function but can rapidly escalate the expression of inflammatory mediators as part of the innate immune response. How the adaptive immune response and other risk factors trigger this process in neurodegeneration have been an exciting topic in research over the last decade (7, 8). A minireview by Candlish and Hefendehl highlight the recent significant advances in this field. In particular, they overview mechanisms that govern the transition of microglia into various subtypes during the neurodegenerative process. A particularly interesting angle is the discussion about the risks that lifestyle factors and aging processes impose on cell phenotype changes. Nitsch et al. then provides a detailed overview on the contribution of microglial signaling to AD pathology with a specific focus on interleukin-23 (IL-23). This paper sheds light on mechanisms by which p40, an IL-23 subunit can be released by microglial cells upon exposure to amyloid-beta (Aβ), a central molecule in AD pathogenesis. One of the key messages of this review is that p40 appears to establish a new link between Aβ and neuroinflammation, possibly via Th17 cells, astrocytes and microglia. Although the identity of effector cells and pathways induced by IL-23 require further elucidation, blocking or neutralizing antibodies for IL-23 may provide promises in reducing cerebral amyloid load or soluble Aβ species (9), which may attract therapeutic interests. Since anti-inflammatory or current antibody treatment approaches have yet to improve the clinical outcome in neurodegeneration, for instance in AD patients (10, 11), the articles in this special issue well serve the purpose of highlighting potential target options for more effective strategies.

The final two articles wrap up the recently revealed aspects of interactions between astrocytes, microglia, peripheral immune cells, and their effect on neuronal networks. Pietrowski et al. reviews the growing evidence of purinergic signaling and its breakdown between glial cells and neurons. This is of particular relevance to non-cell autonomous pathomechanisms in neurodegeneration (1, 7), which can worsen neuronal network function, leading to cognitive or motor decline. This paper also brings up the issue of emerging major transcriptional and functional differences between human and mouse astrocytes and microglia (12, 13), including expression of genes that are a pre-requisite for their interactions with neurons. Considering these potential differences is crucial when interpreting results in mouse disease models that do not entirely recapitulate the human pathobiological phenotype (14). Another timely issue related to glial cell communication concerns immune cells, a topic which has emerged onto the central stage of neurodegeneration research. Copas et al. put this into an interesting perspective. They describe how the genetic risk in Parkinson's Disease (PD) may affect glial cells and conspire with peripheral infections during lifetime, predisposing to a chronic neuroinflammatory response. The authors follow us through the ways infiltrating T cells could play a central role, triggered by antigen-presenting microglia. They argue that this may also lead to altered astrocytic inflammatory responses, and consequently contribute to the loss of dopaminergic neurons. The broad overview of the above disease-related pathways illuminates the role of infection and peripheral immune activation as important risk factors in neurodegenerative diseases.

## Conclusions

Overall, the review articles in this issue remind us of the multiple cell-types that are primarily involved in disease, and also focus on those cell populations that are not innate in the brain. The discussions highlight the need for systems biology approaches to distinguish initiating molecular disturbances that can be obscured by secondary homeostatic responses in many cell populations. Recent examples have already shown us how new technologies and platforms, such as single cell or spatial transcriptomics and human stem cell-based or brain organoids could resolve the above problem (15–17). We anticipate that the emerging data demonstrating human-specific differences in pathogenesis will transform translational science and personalized treatment strategies in this decade.

## Author Contributions

All authors listed have made a substantial, direct and intellectual contribution to the work, and approved it for publication.

## Conflict of Interest

The authors declare that the research was conducted in the absence of any commercial or financial relationships that could be construed as a potential conflict of interest.

## Publisher's Note

All claims expressed in this article are solely those of the authors and do not necessarily represent those of their affiliated organizations, or those of the publisher, the editors and the reviewers. Any product that may be evaluated in this article, or claim that may be made by its manufacturer, is not guaranteed or endorsed by the publisher.
